# *miR-639* promotes the proliferation and invasion of breast cancer cell in vitro

**DOI:** 10.1186/1475-2867-14-39

**Published:** 2014-05-10

**Authors:** Lin Li, Xin-guang Qiu, Peng-wei Lv, Fang Wang

**Affiliations:** 1 Department of Breast Surgey, the First Affiliated Hospital of Zhengzhou University, No. 1, East Jian she Road, Zhengzhou 450052 Henan Province, China

**Keywords:** *miR-639*, Breast cancer, Invasion, Metastases

## Abstract

Breast cancer is characterised by an elevated capacity for tumour invasion and lymph node metastasis, but the cause remains to be determined. Recent studies suggest that microRNAs can regulate the evolution of malignant behaviours by regulating multiple target genes. In this study, we have first confirmed that *miR-639* is up-regulated in metastatic breast cancer tissues and cell line with highly invasive capacity. Furthermore, we provided evidence to demonstrate that up-regulation of *miR-639* contributes breast cancer invasion and metastasis. These data reveal a key role of *miR-639* in breast cancer metastasis and support biological and clinical links between *miR-639* and breast cancer.

## Introduction

Breast cancer-related deaths are caused by cancer metastases rather than the primary tumor. Different subtypes of breast cancer exhibit distinct metastases behaviors in terms of the temporal kinetics and anatomic sites. Estrogen receptor–positive (ER+) breast cancer, predominantly recurs in bone after the diagnosis of the primary tumor [1]. The main mechanisms underlying these observations, however, remain to be elucidated.

MicroRNAs (miRNAs) are small, endogenous, non-coding RNAs which play important gene-regulatory roles in animals via sequence-specific interactions with the 3′UTR of cognate mRNA targets, causing suppression of translation and mRNA decay [2]. It has been firmly established that miRNAs regulate many key cellular processes such as cell growth, differentiation and apoptosis [3,4]. About 50% of annotated human miRNAs are detected in regions of fragile sites, which are associated with cancer. Experiments have confirmed that miRNAs participate in the tumorigenesis progression of many types of cancers, including the breast cancer [5]. Although the number of verified human miRNAs is still expanding, the functions of only a few have been described. Subsets of miRNAs have been identified as potential diagnostic and prognostic markers in malignant tumors [6-8]. *MiR-639* has been reported to have disrupted expression in pathological states [9,10]. Similarly, *miR-639* has been proven to be dysregulated in in serum of patients with bladder cancer. Therefore, the role of *miR-639* in breast cancer caused us a great interest.

In this study, we examined the expression of *miR-639* in breast cancer tissue samples and breast cancer cell lines. We found that *miR-639* levels were up-regulated in metastatic breast cancer tissues and highly invasive cell lines. Furthermore, we have investigated the mechanism of *miR-639* in breast cancer cell lines. These results show that exogenous overexpression of *miR-639* promotes the invasion and migration of breast cancer cells *in vitro*.

## Materials and methods

### Cell culture

MCF-7 cancer cells are breast cancer derived and display an epithelial phenotype and low invasive capacity. MDA-MB-231 (MD231) cancer cells are also breast cancer derived but these cells have a mesenchymal phenotype and high invasive capacity. Both these breast cancer cell lines were obtained from the American Type Culture Collection (ATCC). These cell lines were cultured in DMEM supplemented with 10% heat-inactivated FBS (GIBCO BRL, NY, USA), penicillin (100 units/ml) and streptomycin (100 μg/ml) at 37°C in a humidified 5% CO_2_ atmosphere.

### Tissue samples and reagents

All samples were obtained by surgery and quickly frozen in liquid nitrogen and stored at -80°C. Informed consent was obtained in advance for all patients selected in this study. In parallel, a separate cohort of 84 patients was assembled from a large pool of patients in the database based on histological diagnosis of breast cancer between 2001 and 2005. We retrospectively reviewed the medical records of patients. Total RNAs were extracted from paraffin blocks using the high pure miRNA isolation kit according to the manufacturer’s protocol (Roche, Switzerland) before further analysis.

Both the *miR-639* inhibitor and its mimics were purchased from GenePharma (Shanghai, China). The high pure miRNA isolation kit was purchased from Roche (Basel, Switzerland). The miRcute miRNA qPCR detection kit and miRcute miRNA qPCR detection kit were purchased from TIANGEN BIOTECH (Beijing, China).

### Real-time PCR analysis

Real-time PCR reactions were performed using an ABI 7500 real-time PCR system (Applied Biosystems, CA). Reverse transcription of the extracted miRNA was performed with miRNA-specific primers using the miRcute miRNA first-strand cDNA synthesis kit, and real-time PCR of miRNAs was performed using the miRcute miRNA qPCR detection kit according to the manufacturer’s protocol (TIANGEN BIOTECH, China), the primer is provided by the miRcute miRNA qPCR detection kit.

### Invasion assays and wound-healing experiment

*In vitro* invasion assays, a total of 4 × 10^4^ cells in 200 μl serum-free DMEM medium were plated onto BD BioCoat™ Matrigel™ Invasion Chambers (8 μm pore size; BD Biosciences), and the lower chamber was immediately filled with 500 μl of DMEM medium with 10% FBS as a chemoattractant. After 24 hrs of incubation in a humidified atmosphere containing 5% CO_2_ at 37°C, the membranes were then fixed with methanol and stained by 0.2% crystal violet. For wound-healing experiments, cells were plated in 6-well plates, transfected as indicated, and cultured to confluency. Cells were serum-starved and scraped with a P200 tip (time 0), and pictures (5 fields) were taken at the 24 h time points.

### Informed consent and ethical approval

This study was approved by Institutional Ethic Committee Office of the First Affiliated Hospital of Zhengzhou University. Written informed consent was obtained from the patient for the publication of this article and accompanying images.

### Statistical analysis

The categorical variables were compared among the groups using the *X*-squared test. The continuous variables were analyzed using the two-tailed Student’s *t*-test. A *P* value of <0.05 was considered statistically significant.

## Results

### The association of *miR-639* expression with metastatic rates in patients with breast cancer

**Figure 1:**
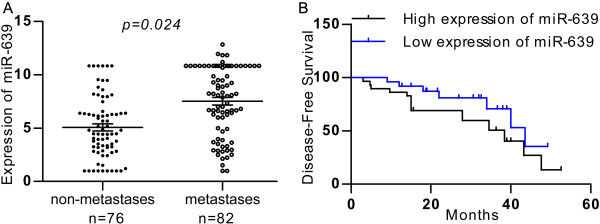
**Clinical association of *miR-639 *with metastases in breast cancer patients. (A)** Mature *miR-639* levels were measured in breast cancer samples by real-time PCR. **(B)** Kaplan-Meier graph representing the probability of disease-free survival in breast cancer patients from the “Milan-INT” dataset stratified. The log-rank test *P* value reflects the significance of the association between low *miR-639* level and disease-free survival.

We first measured mature *miR-639* levels in a group of tissue specimens from breast cancer patients. In the 82 metastatic breast cancer tissues, the expression level of *miR-639* was 2.11 ± 0.31, whereas its expression level in the 76 tissues with non-metastatic breast cancer tissues was 1.19 ± 0.14 (*P = 0.024*; Figure 1A). These results showed that *miR-639* expression levels in primary metastatic breast cancer tissues samples were significantly higher than those non-metastatic breast cancer tissues. The correlations between *miR-639* expression levels and clinical pathological characteristics are summarised in Table 1. Statistically significant reverse associations between *miR-639* expression levels and metastatic rates were observed. In the 74 breast cancer tissues with severe histological signs, the expression level of *miR-639* was 1.73 ± 0.17, which was also significantly higher than the expression level of 1.57 ± 0.23 in 77 tissues with non-histological signs (*P = 0.038*; Table 1).

**Table 1:** **Associtions between *miR-639 *** **and clinical parameters (n = 158)**

**Characteristic**	**No. of patients**		***miR-639 ΔCt***^**a**^	***P***
	**No.**	**%**	**Mean ± SD**	
**Age, years**				
≥60	69	43.7	1.71 ± 0.17	*0.664*
<60	89	56.3	1.64 ± 0.21	
**Pathologe grade**				
I	56	35.4	1.69 ± 0.21	
II	49	31.0	1.86 ± 0.14	*0.089*
III	53	33.5	1.98 ± 0.15	
**T stage**				
**T1**	57	36.1	1.54 ± 0.27	*0.471*
**T2**	50	31.6	1.65 ± 0.24	
**T3**	51	32.3	1.33 ± 0.17	
**M stage**				
M0	76	48.1	1.19 ± 0.14	*0.024*
M1	82	51.9	2.11 ± 0.31	
**Histologic signs of severity** (vascular emboli, perineural invasion, diffuse infiltration)				
None	77	48.7	1.57 ± 0.23	*0.038*
Presence	74	46.8	1.73 ± 0.17	
Missing	7	4.4		
**Smoking history**				
Nonsmoker	111	70.3	1.37 ± 0.23	*0.625*
Smoker	39	24.7	1.47 ± 0.21	
Missing	8	5.1		
**Alcohol history**				
Nondrinker	107	67.7	1.35 ± 0.29	*0.225*
Drinker	42	26.6	1.27 ± 0.36	
Missing	9	5.7		

*Abbreviations*: *SD* standard deviation, *T* tumor stage, *N* lymphnode stage.

^a^ΔCt indicates the difference in the cycle number at which a sample’s fluorescent signal passes a given threshold above baseline (Ct) derived from a specific gene compared with that of *U6* in tumor tissues.

We next analyzed mature *miR-639* levels in the collection of breast cancer patients with clinical characteristics. Patients were divided into two groups with high or low levels of *miR-639*. Remarkably, when tested using the Kaplan-Meier survival analysis, the *miR-639* “low expression” group displayed a significantly longer disease-free survival rates when compared to the “high expression” group (Figure 1B). These data suggest a possible link between *miR-639* expression and breast cancer progression.

### *miR-639* promotes cell proliferation

**Figure 2:**
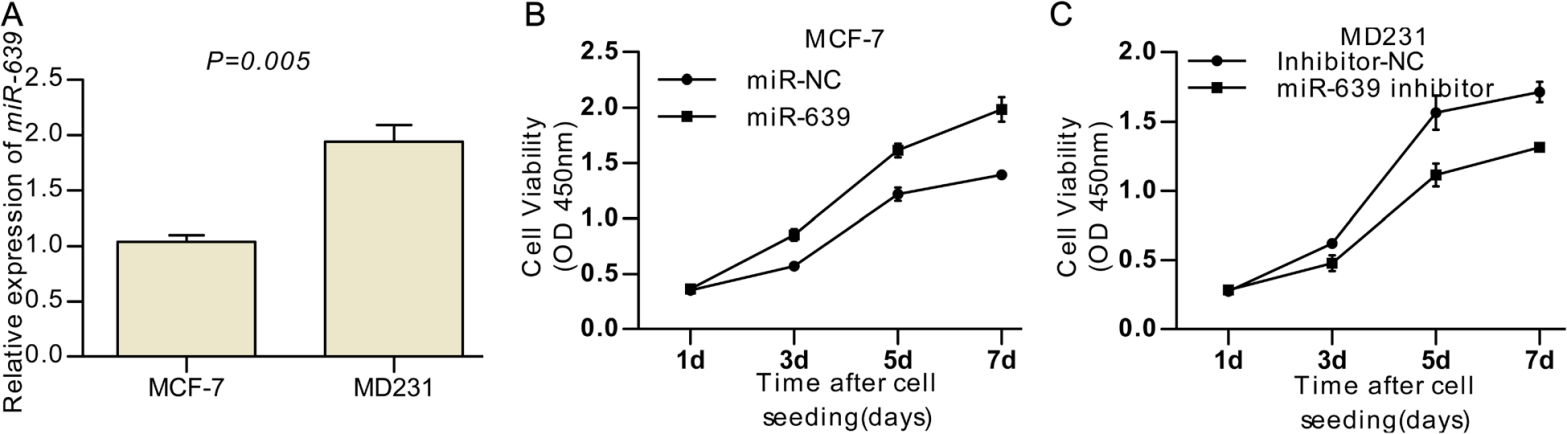
***miR-639 *promotes breast cancer cell proliferation. (A)** Expression levels of *miR-639* in cellular models of metastatic progression were tested by real-time PCR. Values related to the nonmetastatic, less aggressive cell line (MCF-7) are normalised to U6 and shown as the mean and SD. **(B and C)** Cell growth curves: proliferation of phenotypically stable indicated cell lines was monitored by the CCK-8 assay.

MCF-7 cancer cells are breast cancer derived and display an epithelial phenotype and low invasive capacity. MD231 cancer cells are also breast cancer derived but these cells have a mesenchymal phenotype and high invasive capacity and contained a relatively high level of *miR-639* (Figure 2A). First, we assessed the growth of miR-639-transfected and miR-NC-transfected MCF-7 cells after transient transfection. As shown in Figure 2B, *miR-639* was able to increase the proliferation of miR-639-transfected cells compared with miR-NC-transfected cells significantly at day 3 and 5 (P < 0.05, Student’s t-test). We further tested if endogenous expression of *miR-639* was required for breast cancer cell invasion in the higher metastatic cancer cell line MD231. For this purpose, we silenced *miR-639* and this treatment led to an approximately 2-fold decline in growth properties (Figure 2C).

### Ectopic expression of *miR-639* promotes cancer metastasis

**Figure 3:**
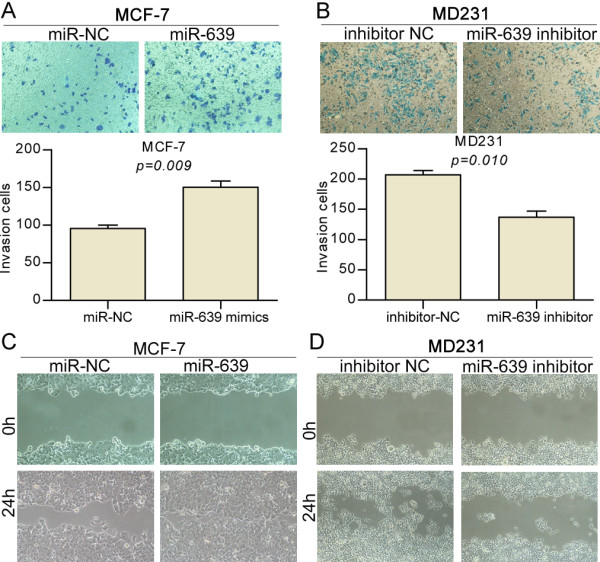
***miR-639 *promotes cell invasion and migration. (A and B)** Representative pictures of cells migrated through the filter, stained with crystal violet, and taken at the same magnification and absolute quantifications as cells that had invaded through the transwell. **(C and D)** Wound-healing assay showing that gain of *miR-639* promotes cell migration and loss of *miR-639* supresses cell migration.

In light of the preceding data, we aimed to determine more directly if *miR-639* plays a causal role in the aggressive traits of breast cancer cells. MCF-7 cancer cells display an epithelial phenotype and low invasive capacity and MD231 cancer cells have a mesenchymal phenotype and high invasive capacity. We used these cell lines to investigate how gain or loss of function of *miR-639* impacted cell migration and invasion, which are hallmarks of metastatic capacity. The MD231 cells displayed high migration capacities and contained a relatively high level of *miR-639* (Figure 2A). In the transwell assays shown in Figure 3B, down regulation of *miR-639* in MD231 cells decreased invasive abilities 3-fold compared to the same cells expressing miR-NC. We further tested if endogenous expression of *miR-639* was required for cell invasion in the lower metastatic cancer cell line MCF-7. For this purpose, we upregulated *miR-639* and this treatment led to an approximately 2-fold augmentation in invasive properties (Figure 3A). Furthermore, the pro-migration effects of *miR-639* were observed in wound-healing assays in MCF-7 and MD231 cells (Figure 3C and D).

## Discussion

Although a global reduction of miRNA abundance appears to be a general trait of human cancers, playing a causal role in the metastatic phenotype [11-13], several miRNAs are up-regulated in tumors [14], Recently, miRNAs have been shown to be related to tumor metastasis [15], providing a new perspective on the metastatic process. Nonetheless, The role of miRNAs in breast cancer has been widely investigated. Here, we will focus on miRNA-639 promotes breast cancer metastasis.

In this study, we described for the first time the *miR-639* is markly upregulated in metastatic breast cancer in large samples. We also found that *miR-639* is closely related to the cancer metastasis and *miR-639* “high expression” group displayed a significantly poorer disease-free survival rates. We have proven that the down-regulation of *miR-639* is crucial in breast cancer metastasis and demonstrated that *miR-639* acts as a putative oncogene. MD231 cells, which stably express *miR-639* ectopically, were transiently transfected with the *miR-639 inhibitor*, and MCF-7 cells were transfected with *miR-639 mimics*. This could be in line with the internal environment of the cells.

The genetic and epigenetic silencing of tumor suppressor genes is considered a vital molecular event in the development and progression of breast cancer [16]. This study first proves that *miR-639* is up-regulated in metastatic breast cancer. Aberrant patterns of miRNA expression are implicated in human diseases including breast cancer. Recent studies have identified miRNAs regulated by estrogens in human breast cancer cells, human endometrial stromal and myometrial smooth muscle cells, rat mammary gland, and mouse uterus. The decline of estradiol levels in postmenopausal women has been implicated in various age-associated disorders [17]. The role of estrogen- regulated *miR-639* expression has yet to be explored. As miRNAs function mainly through the inhibition of target genes and Wu et al. [18] showed p21Cip1/Waf1 expression was reduced by *miR-639*. This result may preliminary explain the mechanism of *miR-639*.

## Conclusion

In conclusion, our results have proven that *miR-639* plays a causal role in the metastases of breast cancer. These findings have implications for understanding the mechanism of breast cancer metastasis, and *miR-639* may be a valuable maker and target for prevention or adjuvant therapy in breast cancer patients.

## Competing interests

The authors declare no competing financial interests.

## Authors’ contribution

Guarantor of integrity of the entire study: XQ, LL. Study concepts and design: LL. Literature research: PL. Cancer cell and molecular studies: LL and FW. Experimental studies/data analysis: LL and PL. Statistical analysis and manuscript preparation: LL. Manuscript editing: XQ. All authors read and approved the final manuscript.
